# The varied roles of *pilA-N, omcE, omcS, omcT*, and *omcZ* in extracellular electron transfer by *Geobacter sulfurreducens*

**DOI:** 10.3389/fmicb.2023.1251346

**Published:** 2023-10-10

**Authors:** Jie Jiang, Pengchen He, Ying Luo, Zhaofeng Peng, Yongguang Jiang, Yidan Hu, Lei Qi, Xiuzhu Dong, Yiran Dong, Liang Shi

**Affiliations:** ^1^School of Environmental Studies, China University of Geosciences-Wuhan, Wuhan, China; ^2^State Key Laboratory of Microbial Resources, Institute of Microbiology, Chinese Academy of Sciences, Beijing, China; ^3^State Key Laboratory of Biogeology and Environmental Geology, China University of Geosciences-Wuhan, Wuhan, China; ^4^State Environmental Protection Key Laboratory of Source Apportionment and Control of Aquatic Pollution, Ministry of Ecology and Environment, China University of Geosciences-Wuhan, Wuhan, China; ^5^Hubei Key Laboratory of Yangtze Catchment Environmental Aquatic Science, China University of Geosciences-Wuhan, Wuhan, China

**Keywords:** *pilA-N*, multiheme *c-*type cytochromes, extracellular electron transfer, biofilms, *Geobacter metallireducens*, *Geobacter sulfurreducens*

## Abstract

*Geobacter sulfurreducens* mediates extracellular electron transfer (EET) reactions with different substrates, such as solid-phase Fe(III)-containing minerals, anodes and the cells of *Geobacter metallireducens*. To compare their roles in EET, the *pilA-N, omcE*, *omcS*, *omcT* and *omcZ* genes of *G. sulfurreducens* were systematically deleted. All mutants showed impaired and varied ability to form biofilms on nonconductive surface. Deletion of *omcE* also impaired bacterial ability to reduce ferrihydrite, but its impacts on the ability for anode reduction and the co-culture of *G. metallireducens*-*G. sulfurreducens* were minimal. The mutant without *omcS* showed diminished ability to reduce ferrihydrite and to form the co-culture, but was able to regain its ability to reduce anodes. Deletion of *omcT*, *omcZ* or *pilA-N* alone impaired bacterial ability to reduce ferrihydrite and anodes and to form the co-culture. Deletion of all tested genes abolished bacterial ability to reduce ferrihydrite and anodes. Triple-deletion of all *omcS, omcT* and *omcZ* abolished the ability of *G. sulfurreducens* to co-culture with *G. metallireducens*. However, deletion of only *omcZ* or *pilA-N* or both *omcS* and *omcT* abolished the ability of *G. sulfurreducens* without hydrogenase gene *hybL* to co-culture with *G. metallireducens*, which show their indispensable roles in direct electron transfer from *G. metallireducens* to *G. sulfurreducens*. Thus, the roles of *pilA-N, omcE*, *omcS*, *omcT* and *omcZ* for *G. sulfurreducens* in EET vary substantially, which also suggest that possession of PilA-N and multiple cytochromes of different structures enables *G. sulfurreducens* to mediate EET reactions efficiently with substrates of different properties.

## Introduction

The dissimilatory Fe(III)-reducing bacterium *Geobacter sulfurreducens* possesses extracellular electron transfer (EET) capability, through which *G. sulfurreducens* can respire on solid-phase Fe(III) oxides and anodes and form syntrophic co-culture with *Geobacter metallireducens* (Caccavo et al., 1994; Bond and Lovley, 2003; Summers et al., 2010). In latter, *G. metallireducens* oxidizes ethanol and then directly transfers the electrons released from ethanol oxidation to *G. sulfurreducens* (i.e., direct interspecies electron transfer or DIET). *G. sulfurreducens* subsequently uses the received electrons to reduce fumarate (Summers et al., 2010). The pilin protein PilA-N is crucial to the EET capability of *G. sulfurreducens* and was believed to form the conductive nanowire to mediate EET directly (Reguera et al., 2005; Richter et al., 2009; Summers et al., 2010). In addition to DIET, H_2_-mediated interspecies electron transfer also occurs from *G. metallireducens* to *G. sulfurreducens* during their co-culture (Summers et al., 2010).

Recent structural analyses, however, revealed that the PilA-N and PilA-C of *G. sulfurreducens* formed a filamentous structure that was unstable extracellularly and electrically nonconductive (Gu et al., 2021; Wang et al., 2022b,c). Instead, the nanowire made of PilA-N and PilA-C was proposed to be responsible for extracellular secretion of multiheme *c*-type cytochromes (*c*-Cyts) OmcS and OmcZ that formed conductive filaments, respectively (Filman et al., 2019; Wang et al., 2019; Yalcin et al., 2020; Yalcin and Malvankar, 2020; Gu et al., 2021; Wang et al., 2022a,b; Gu et al., 2023). Similarly, the multiheme *c*-Cyt OmcE of *G. sulfurreducens* also formed the conductive nanowire extracellularly (Wang et al., 2022c). Notably, the structures of these proteins vary substantially (Filman et al., 2019; Wang et al., 2019; Yalcin et al., 2020; Yalcin and Malvankar, 2020; Gu et al., 2021; Wang et al., 2022a,b,c; Gu et al., 2023).

Similar to PilA-N, OmcS, OmcT (an OmcS homolog) and OmcE are involved in extracellular reduction of metal oxides by *G. sulfurreducens* (Lovley et al., 2004; Mehta et al., 2005; Reguera et al., 2005; Shi et al., 2007), OmcZ is involved in extracellular reduction of anodes (Nevin et al., 2009; Richter et al., 2009; Inoue et al., 2011; Richter et al., 2012) and OmcS is involved in the co-culture of *G. metallireducens*-*G. sulfurreducens* (Summers et al., 2010). Compared to that of PilA-N and OmcZ, the roles of OmcE and OmcS in extracellular reduction of anodes are trivial (Nevin et al., 2009; Richter et al., 2009). In addition to their roles in EET, PilA-N and OmcZ also play non-conductive roles in biofilm formation (Reguera et al., 2006; Richter et al., 2009). However, our current understanding of the roles of PilA-N, OmcE, OmcS, OmcT and OmcZ for *G. sulfurreducens* in EET is still far from complete. For instance, the roles of OmcT, OmcE and OmcZ in formation of co-culture with *G. metallireducens* are unknown.

In this investigation, we systemically compared the roles of *pilA-N*, *omcE*, *omcS*, *omcT* and *omcZ* genes of *G. sulfurreducens* in EET as well as biofilm formation. Our results showed that their roles in biofilm formation, extracellular reductions of Fe(III) oxides and anodes and the co-culture of *G. metallireducens*-*G. sulfurreducens* varied substantially.

## Experimental procedures

### Bacterial strains and cultivation conditions

Both *Geobacter metallireducens* GS-15 (ATCC^®^ 53774^™^) and *Geobacter sulfurreducens* PCA (ATCC^®^ 51573^™^) were purchased from American Type Culture Collection (Manassas, VA, United States) (Supplementary Table S1). *G. metallireducens* was routinely cultured in the anaerobic NB medium (0.38 g/L KCl, 0.2 g/L NH_4_Cl, 0.069 g/L NaH_2_PO_4_·H_2_O, 0.04 g/L CaCl_2_·2H_2_O, 0.2 g/L MgSO_4_· 7H_2_O, 1% [vol/vol] trace mineral mix) with 20 mM acetate as the electron donor and 55 mM Fe(III)-citrate as the electron acceptor. The pH was adjusted to 6.8 with 2 g/L NaHCO_3_ and degassed with 80% N_2_ and 20% CO_2_ (Levar et al., 2017). *G. sulfurreducens* was also routinely cultured in the anaerobic NB medium with 20 mM acetate and 40 mM fumarate as the sole electron donor and receptor, respectively.

### Construction of gene-deletion mutants of *Geobacter sulfurreducens*

The gene-deletion mutants were constructed by following the procedures described previously (Coppi et al., 2001; Marx and Lidstrom, 2002; Lloyd et al., 2003; Liu et al., 2014, 2015). All constructed mutants were verified by PCR and DNA sequencing. To complement the mutants, the respective genes were cloned separately into the plasmid pBBR1MCS-5 that was maintained in *Escherichia coli* DH5α (Kovach et al., 1995) (Supplementary Tables S1, S2). The constructs were verified by DNA sequencing and then transformed into their respective mutants. As controls, the mutants were also transformed with empty vector pBBR1MCS-5. All gene-deletion mutants and plasmid constructs made and the oligonucleotide primers used in this investigation are listed in Supplementary Tables S1–S3, respectively.

### Growth with fumarate and Fe(III) reductions

All constructed mutants were tested for their ability to grow with fumarate as the terminal electron acceptor and to reduce Fe(III)-citrate and ferrihydrite. Fumarate and Fe(III)-citrate were purchased from Sinopharm Chemical Reagent Co., Ltd. (Shanghai, China). Ferrihydrite was prepared and characterized as described before (Schwertmann and Cornell, 2000; Guo et al., 2022). Growth with fumarate and reductions of Fe(III)-citrate and ferrihydrite were carried out by following the previously established procedures (Liu et al., 2014, 2015).

### Biofilm formation and anode reductions

All constructed mutants were also tested for their ability to form biofilms and to reduce anodes. The procedures for measuring biofilm formation with crystal violet staining and electricity production by monitoring output voltages from microbial fuel cells (MFC) were described before (Wang et al., 2023). Briefly, MFCs of dual-chambers were used in this study. PS2024V multi-channel data acquisition unit (SMACQ, Beijing, China) was used to monitor bacterial growth on the anodes and electricity outputs. At the end of experiments, a Leica TCS SP8 MP multiphoton confocal microscope (Wetzlar, Germany) was used to examine the biofilms formed on the anodes.

All experimental procedures were carried in an anaerobic chamber (Coy Laboratory Products, Inc., Grass Lake, MI, United States) with 80% N_2_ and 20% CO_2_.

*Co-culture.* The wild-type (WT) of *G. sulfurreducens* and its mutants were co-cultured with *G. metallireducens* in the anaerobic NB medium with 20 mM ethanol as the electron donor and 40 mM fumarate as the terminal electron acceptor (Summers et al., 2010). At the beginning of the first generation of co-culture, the cell densities used were 3.9 × 10^5^ cells/mL for *G. metallireducens* and 7.7 × 10^5^ cells/mL for the WT and mutants of *G. sulfurreducens*. Exponential phase of the cells from the first generation of co-cultures were used for the second generation of co-culture at initial cell densities of ~2.5 × 10^6^ copies/mL *Geobacter* 16S RNA genes. The levels of ethanol, fumarate, malate and succinate during co-culture were monitored with a LC-20A high performance liquid chromatography (HPLC) that was equipped with a SPD-M20A UV detector and a RID-20A high-sensitivity refractive index detector (Shimadzu, Kyoto, Japan). Supplementary Figure S1 shows metabolisms of ethanol, fumarate, malate and succinate between these *Geobacte*r species during co-culture. The chemical compounds were separated with an Aminex NPX-87H column (Bio-Rad Laboratories, Shanghai, China) (Summers et al., 2010; Shi et al., 2022). Ethanol, malate and succinate were purchased from Sinopharm Chemical Reagent Co., Ltd. Quantitative PCR (qPCR) was used to monitor bacterial growth during co-culture (QuantStudio3, Thermo Fisher Scientific China Co., Ltd., Shanghai, China) (Summers et al., 2010). The plasmids and the oligonucleotide primers used in qPCR are listed in Supplementary Tables S1, S2.

### Statistical analyses

All values are expressed as means ± standard deviations. Student’s *t* test was used for comparing groups.

## Results

### Gene-deletion mutants

To investigate their roles in EET, the genes for PilA-N, OmcE, OmcS, OmcT and OmcZ of *G. sulfurreducens* were systematically deleted. A total of nine gene-deletion mutants were constructed in this investigation, which included five single-gene-deletion mutants *ΔomcS, ΔomcT, ΔomcZ, ΔomcE* and *ΔpilA-N*; a double-gene-deletion mutant *ΔomcSΔomcT*; a triple-gene-deletion mutant *ΔomcSΔomcTΔomcZ*; a quadruple-gene-deletion mutant *ΔomcSΔomcTΔomcZΔomcE* and a mutant defective in all investigated genes (i.e., *ΔomcSΔomcTΔomcZΔomcEΔpilA-N*) (Supplementary Table S1).

### Growth with fumarate and Fe(III) reductions

**Figure 1:**
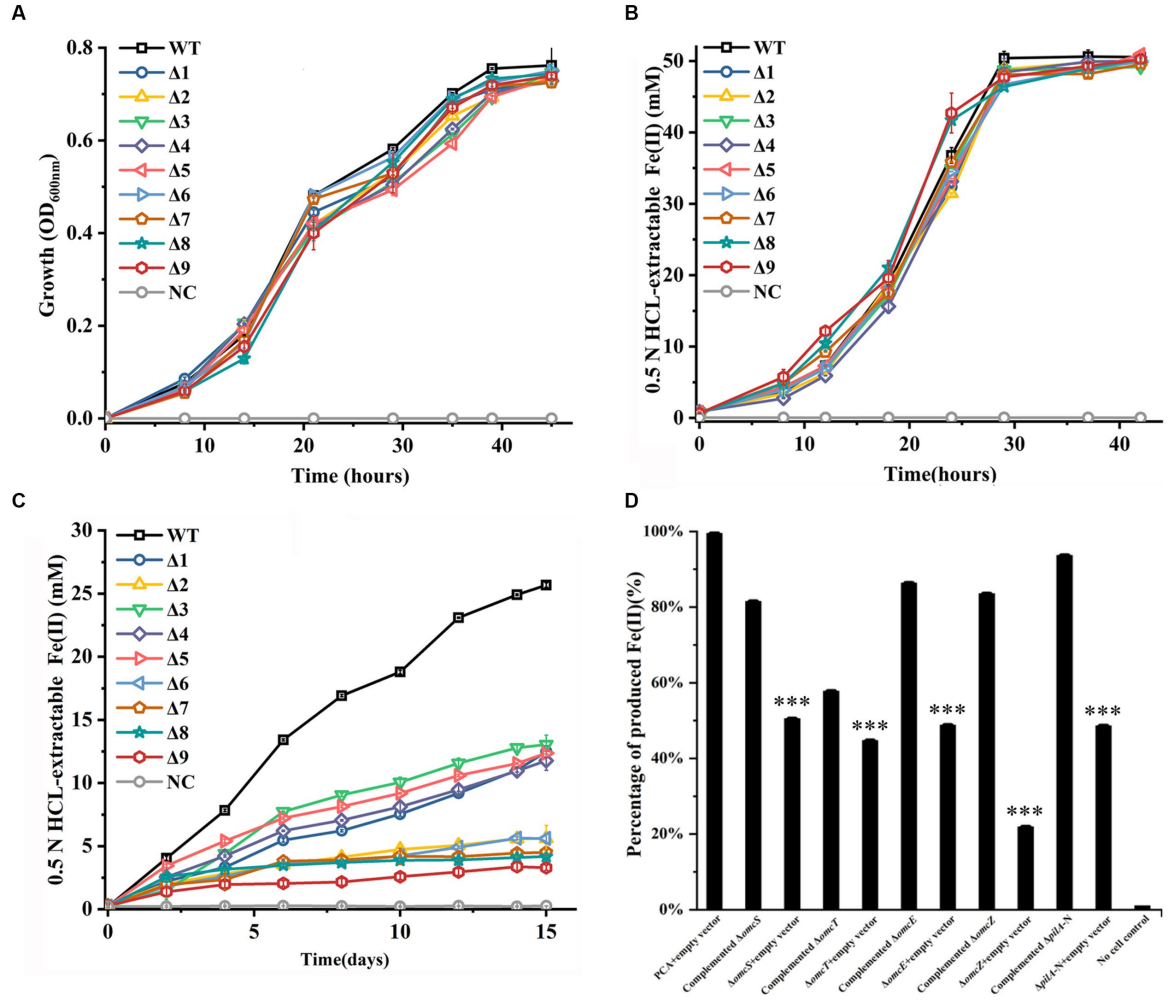
Growth with fumarate and reductions of Fe(III)-citrate and ferrihydrite by the constructed mutants of *Geobacter sulfurreducens*. **(A)** Growth with fumarate as the sole terminal electron acceptor. **(B)** Fe(III)-citrate reduction. **(C)** Ferrihydrite reduction over 15 days. **(D)** Complement of single-gene-deletion mutants for ferrihydrite reduction. The reductions were carried over 15 days. The shown are those at 15 days after reduction. WT, wild-type of *G. sulfurreducens*; Δ1, Δ*omcS*; Δ2, Δ*omcT*; Δ3, Δ*omcE*; Δ4, Δ*omcZ*; Δ5, Δ*pilA-N*; Δ6, Δ*omcS*Δ*omcT*; Δ7, Δ*omcS*Δ*omcT*Δ*omcZ*; Δ8, Δ*omcS*Δ*omcT*Δ*omcZ*Δ*omcE*; Δ9, Δ*omcS*Δ*omcT*Δ*omcZ*Δ*omcE*Δ*pilA-N*; NC, no cell control. The values plotted at each time point are the average OD_600_ **(A)** and 0.5 N HCl extractable Fe(II) **(B,D)** measured for each strain (*n* = 3), respectively, and error bars are standard deviations. For points with no error bar, the error was smaller than the size of the symbol. In **(D)**, the results are reported as the percentage of activity relative to that observed with PCA + empty vector and standard error of the mean (*n* = 3). Student’s *t* test was used for comparing complement strains and their respective control groups. ***, *p* ≤ 0.001.

All constructed mutants were tested for their ability to grow with fumarate and to reduce Fe(III)-citrate and ferrihydrite. Similar to the WT of *G. sulfurreducens*, all mutants showed no apparent deficiency in growth on fumarate (Figure 1A) or Fe(III)-citrate reduction (Figure 1B). However, all mutant displayed diminished ability to reduce ferrihydrite under the condition tested, as compared to that of WT (Figure 1C). At 15 days after reduction of ferrihydrite, the amounts of Fe(II) formed by all tested mutants were significantly lower than that by WT (Figure 1C; Supplementary Table S4). Furthermore, the more gene deleted, the less Fe(II) formed (Figure 1C; Supplementary Table S4).

The single-gene-deletion mutants were complemented with their respective genes. Empty vector was also introduced into these mutants and WT as controls. The complement and control strains were all tested for ferrihydrite reduction over 15 days. Figure 1D shows the results at 15 days after reduction. Compared to those with empty vector, the single-gene-deletion mutants complemented with their respective gene all showed improved ability to reduce ferrihydrite, which indicated that the gene deletions were nonpolar.

### Biofilm formation and anode reduction

**Figure 2:**
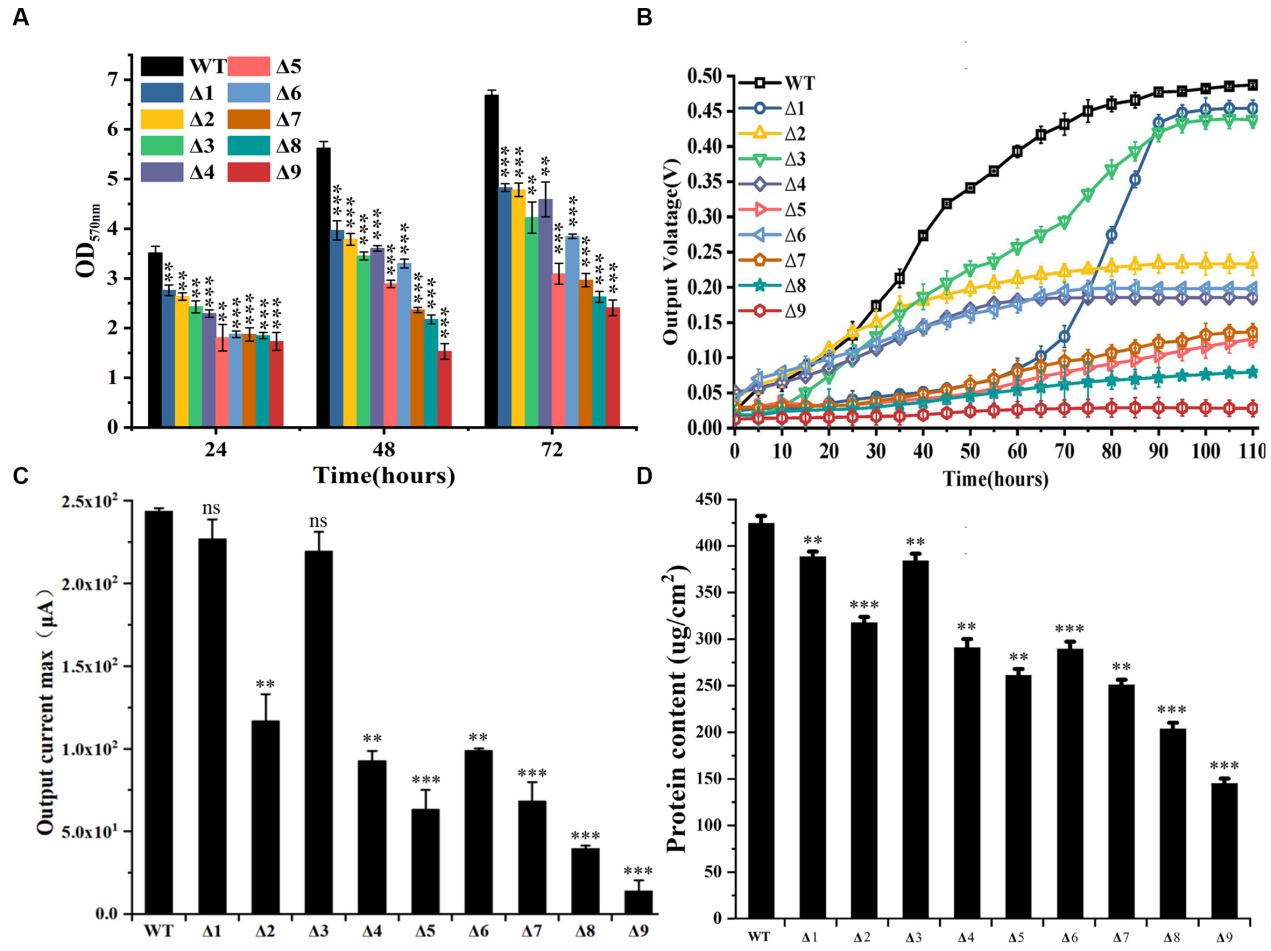
Biofilm formation and anode reduction by the constructed mutants of *Geobacter sulfurreducens*. **(A)** Absorbance of crystal violet (OD_565_) extracted from stained biofilms grown on non-conductive surfaces (*n* = 3). **(B)**. Output voltage of bacterial strains grown on anodes over 110 h. The values plotted at each time point are the average output voltage measured for each strain from triplicate assays and error bars are standard deviations. For points with no error bar, the error was smaller than the size of the symbol. **(C)** Maximum output current of bacterial strains grown on anodes at 110 h (*n* = 3). **(D)**. Bacterial protein contents on anodes at 110 h after growth (*n* = 3). WT, wild-type of *G. sulfurreducens*; Δ1, Δ*omcS*; Δ2, Δ*omcT*; Δ3, Δ*omcE*; Δ4, Δ*omcZ*; Δ5, Δ*pilA-N*; Δ6, Δ*omcS*Δ*omcT*; Δ7, Δ*omcS*Δ*omcT*Δ*omcZ*; Δ8, Δ*omcS*Δ*omcT*Δ*omcZ*Δ*omcE*; Δ9, Δ*omcS*Δ*omcT*Δ*omcZ*Δ*omcE*Δ*pilA-N*. In **(A,C,D)**, Student’s *t* test was used for comparing WT and the mutants. ns, *p* > 0.05; *, *p* ≤ 0.05; **, *p* ≤ 0.01; ***, *p* ≤ 0.001.

Compared to WT, all mutants exhibited reduced ability to form biofilms on the cell culture plates that were not electrically conductive over 72 h after incubation (Figure 2A). At 72 h after incubation, the absorbance of crystal violet (OD_565_) extracted from stained biofilms grown on non-conductive surfaces by the WT was higher than that of all tested mutants. Furthermore, the OD_565_ of Δ*pilA-N* was lower than that of Δ*omcS*, Δ*omcT*, Δ*omcZ*, Δ*omcE* or Δ*omcS*Δ*omcT*, but was similar to that of Δ*omcS*Δ*omcT*Δ*omcZ* or Δ*omcS*Δ*omcT*Δ*omcZ*Δ*omcE* (Figure 2A; Supplementary Table S5). All mutants also exhibited reduced ability to grow on the anode surfaces, as compared to WT. Notably, Δ*omcS* grew very slowly at beginning, as measured by its output voltage on anodes. At 60 h after growth, its output voltage increased substantially and then plateaued at 85 h after growth (Figure 2B). At 110 h after growth, the measured maximum output currents of Δ*omcS* and Δ*omcE* on anodes were similar to that of WT, but significantly higher than that of the rest of mutants (Figure 2C; Supplementary Table S6). Additionally, the maximum output current of *ΔpilA-N* was lower than that of Δ*omcT*, Δ*omcZ* and Δ*omcS*Δ*omcT*, but was similar to that of other mutants deficient in >2 genes (Figure 2C; Supplementary Table S6). Similar phenomena were also observed on the ability of WT and tested mutants to form biofilms on the anodes (Figure 2D; Supplementary Figure S2).

### Co-culture

**Figure 3:**
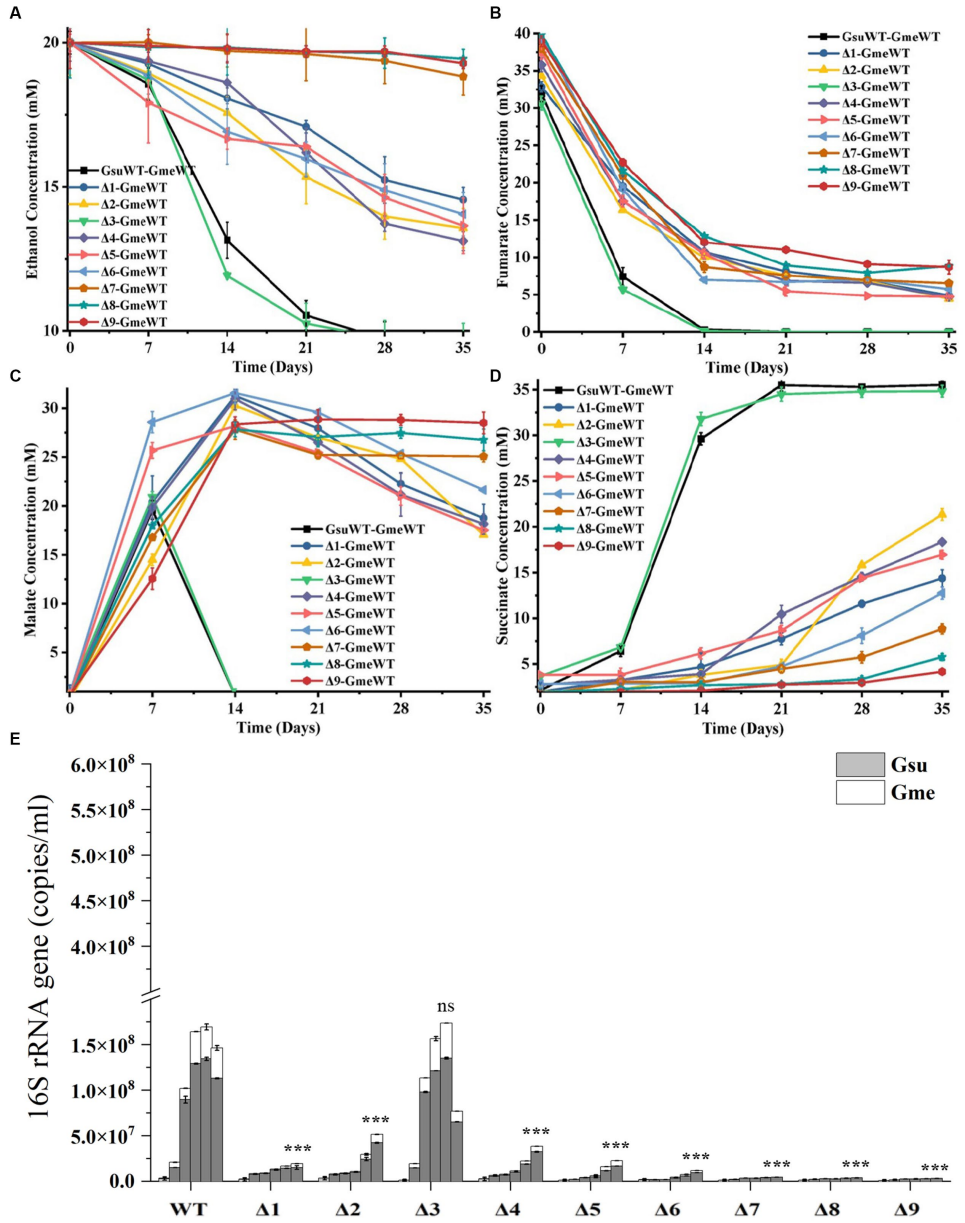
The second generation of co-cultures in the presence of *hybL* gene of *Geobacter sulfurreducens* over 35 days. The values plotted at each time point are the average ethanol **(A)**, fumarate **(B)**, malate **(C)** and succinate **(D)** measured for each strain (*n* = 3), respectively, and error bars are standard deviations. For points with no error bar, the error was smaller than the size of the symbol. **(E)** The copies of combined 16S rRNA genes of *Geobacter metallireducens* (Gme) and *G. sulfurreducens* (Gsu) (*n* = 3). The predetermined time points for sample collections in **(E)** are the same to those in **(A–D)**. WT, wild-type of *G. sulfurreducens* or *G. metallireducens*; Δ1, Δ*omcS* of *G. sulfurreducens*; Δ2, Δ*omcT* of *G. sulfurreducens*; Δ3, Δ*omcE* of *G. sulfurreducens*; Δ4, Δ*omcZ* of *G. sulfurreducens*; Δ5, Δ*pilA-N* of *G. sulfurreducens*; Δ6, Δ*omcS*Δ*omcT* of *G. sulfurreducens*; Δ7, Δ*omcS*Δ*omcT*Δ*omcZ* of *G. sulfurreducens*; Δ8, Δ*omcS*Δ*omcT*Δ*omcZ*Δ*omcE* of *G. sulfurreducens*; Δ9, Δ*omcS*Δ*omcT*Δ*omcZ*Δ*omcE*Δ*pilA-N* of *G. sulfurreducens*. In **(E)**, Student’s *t* test was used for comparing the maximum copies of combined 16S rRNA genes of Gme and Gus from WT and that of the mutants. ns, *p* > 0.05; ***, *p* ≤ 0.001.

During the first and second generations of the co-cultures between *G. metallireducens* and the WT or gene-deletion mutants of *G.sulfurreducens*, substantial differences in the metabolisms of ethanol, fumarate, malate and succinate as well as the copy numbers of combined bacterial 16S rRNA genes were detected between WT and other mutants, with the exception of Δ*omcE*. Compared to that of the first generation (Supplementary Figures S3A–E), these differences between that of WT and Δ*omcE* and that of other mutants were much more pronounced in the second generation of the co-cultures (Figures 3A–E). Furthermore, these differences in decreases of ethanol (Figure 3A) and fumarate (Figure 3B), changes of malate (Figure 3C) and increases of succinate (Figure 3D) and the copy numbers of combined 16S rRNA genes (Figure 3E) were similar with each other. The maximum copy numbers of combined 16S rRNA genes detected at 35 days after the second generation of co-cultures between *G. metallireducens* and the WT were similar to that between *G. metallireducens* and Δ*omcE*, but were much higher than that between *G. metallireducens* and the rest of mutants (Figure 3E; Supplementary Table S7). In latter, the more genes deleted; the less copy numbers of combined 16S rRNA genes detected (Figure 3E; Supplementary Table S6).Similar phenomena were also observed in the levels of succinate detected in 35 days after the second generation of co-cultures (Figure 3D).

**Figure 4:**
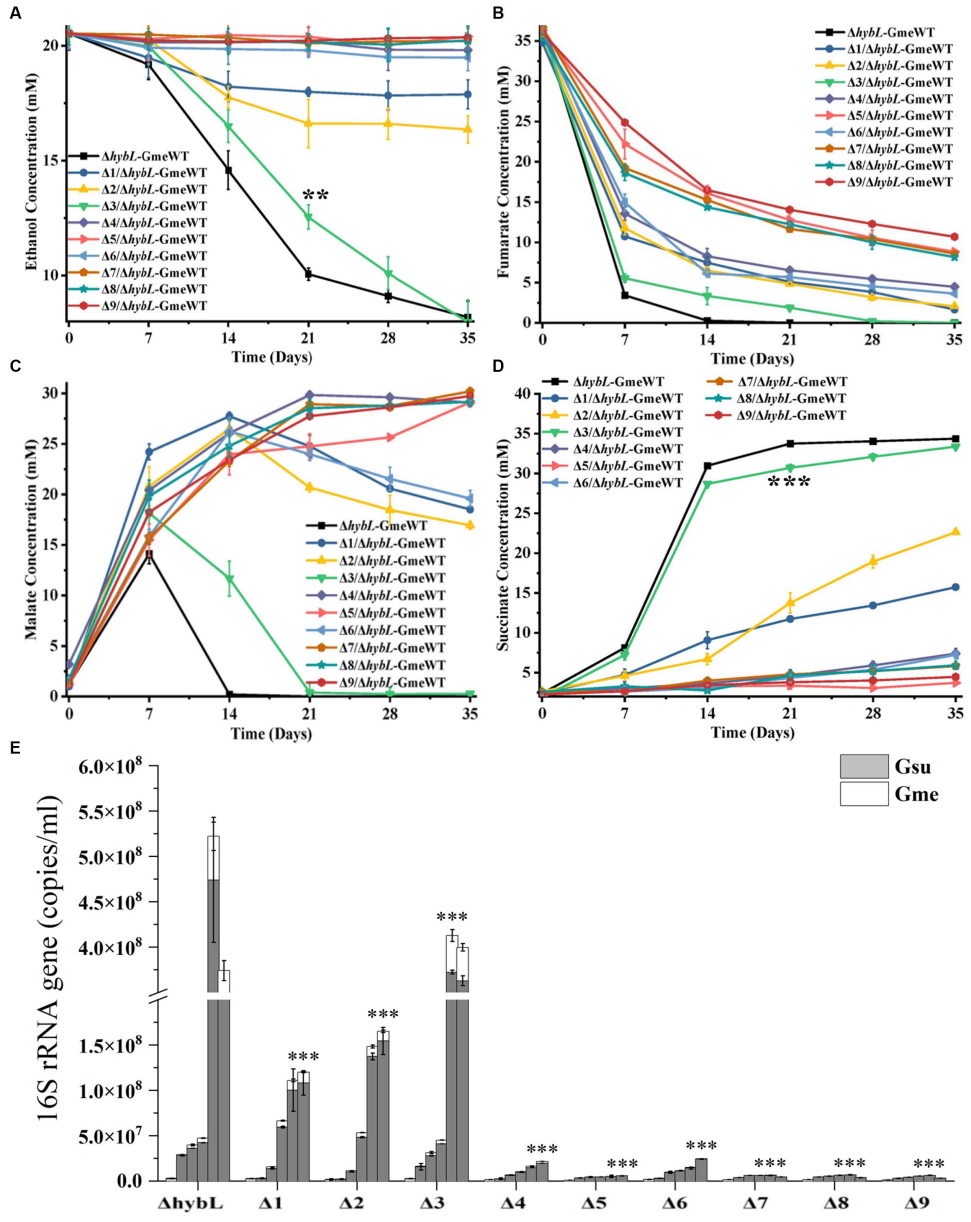
The second generation of co-cultures in the absence of *hybL* gene of *Geobacter sulfurreducens* over 35 days. The values plotted at each time point are the average ethanol **(A)**, fumarate **(B)**, malate **(C)** and succinate **(D)** measured for each strain (n = 3), respectively, and error bars are standard deviations. For points with no error bar, the error was smaller than the size of the symbol. **(E)** The copies of combined 16S rRNA genes of *Geobacter metallireducens* (Gme) and *G. sulfurreducens* (Gsu) (*n* = 3). The predetermined time points for sample collections in **(E)** are the same to those in **(A–D)**. WT, wild-type of *G. metallireducens*; Δ*hybL*, Δ*hybL* of *G. sulfurreducens*; Δ1/Δ*hybL*, Δ*omcS* of Δ*hybL*; Δ2/Δ*hybL*, Δ*omcT* of Δ*hybL*; Δ3/Δ*hybL*, Δ*omcE* of Δ*hybL*; Δ4/Δ*hybL*, Δ*omcZ* of Δ*hybL*; Δ5/Δ*hybL*, Δ*pilA-N* of Δ*hybL*; Δ6/Δ*hybL*, Δ*omcS*Δ*omcT* of Δ*hybL*; Δ7/Δ*hybL*, Δ*omcS*Δ*omcT*Δ*omcZ* of Δ*hybL*; Δ8/Δ*hybL*, Δ*omcS*Δ*omcT*Δ*omcZ*Δ*omcE* of Δ*hybL*; Δ9/Δ*hybL*, Δ*omcS*Δ*omcT*Δ*omcZ*Δ*omcE*Δ*pilA-N* of Δ*hybL*. In **(A,D,E)** student’s *t* tests were used for comparing the ethanol and succinate levels at 21 days after growth between the co-culture of Gme and Gus from *ΔhybL* and that of *ΔomcE/ΔhybL* **(A,D)** and the maximum copies of combined 16S rRNA genes of Gme and Gus from *ΔhybL* and that of other mutants without *hybL* **(E)**. **, *p* ≤ 0.01; ***, *p* ≤ 0.001.

Previous results suggested the involvement of H_2_ in the interspecies electron transfer during the co-culture of *G. metallireducens* and *G. sulfurreducens* as deletion of the hydrogenase gene *hybL* of *G. sulfurreducens* improved the bacterial ability to form culture with *G. metallireducens* (Summers et al., 2010). To investigate the roles of *pilA-N*, *omcE*, *omcS*, *omcT* and *omcZ* of *G. sulfurreducens* in the interspecies electron transfer during the co-culture with *G. metallireducens* in the absence of H_2_-mediated interspecies electron transfer, we further deleted the *hybL* gene in the mutants described above (Supplementary Table S1). No apparent differences between the Δ*hybL* and the mutants without *hybL* were observed in their ability to grow with fumarate and to reduce Fe(III)-citrate (Supplementary Figures S4A,B). Similar to previous observations (Summers et al., 2010), deletion of *hybL* increased the growth of the first and second generations of co-culture between Δ*hybL* of *G. sulfurreducens* and *G. metallireducens* (Supplementary Figures S5A–E; Figures 4A–E). In the absence of *hybL*, differences between Δ*hybL* and all the mutants tested were observed in the metabolisms of ethanol, fumarate, malate and succinate as well as the maximum copy numbers of combined bacterial 16S rRNA genes during the first and second generations of the co-culture (Supplementary Figures S5A–E; Figures 4A–E). In 35 days after the second generation of co-culture, no or little growth was observed between *ΔomcZ/*Δ*hybL, ΔpilA-N/*Δ*hybL, ΔomcSΔomcT/*Δ*hybL*, *ΔomcSΔomcTΔomcZ/*Δ*hybL, ΔomcSΔomcTΔomcZΔomcE/*Δ*hybL or ΔomcSΔomcTΔomcZΔomcEΔpilA-N/*Δ*hybL and G. metallireducens*. Furthermore, the maximum copy numbers of combined 16S rRNA genes detected in the second generation of co-cultures decreased in the order of Δ*hybL* > *omcE/*Δ*hybL > ΔomcT/*Δ*hybL > ΔomcS /*Δ*hybL*, which showed that *omcE/*Δ*hybL* displayed a modest decrease (*p* < 0.001) in its ability to form co-culture with *G. metallireducens* as compared to that of Δ*hybL* (Figure 4E; Supplementary Table S8).

## Discussions

Deletions of *pilA-N, omcE, omcS, omcT* or/and *omcZ* of *G. sulfurreducens* had no impact on the bacterial growth on fumarate and reduction of Fe(III)-citrate, which are consistent with previous observations (Mehta et al., 2005; Reguera et al., 2005; Nevin et al., 2009; Summers et al., 2010). Previous results also showed the involvements of the *pilA-N, omcE, omcS* and *omcT* in reduction of solid-phase Fe(III) oxide (Mehta et al., 2005; Reguera et al., 2005). Consistent with these previous results, the Δ*pilA-N*, Δ*omcE*, Δ*omcS*, and Δ*omcT* prepared in this investigation showed the impaired ability to reduce solid-phase ferrihydrite under the condition tested, as compared to the WT. However, it should be noted that *omcE* was only involved in the initial stage of Fe(III) oxide reduction and the role of *omcT* in Fe(III) oxide reduction was via *omcS* (Mehta et al., 2005). Furthermore, our results also showed the diminished ability of *omcZ* in ferrihydrite reduction under the conditions tested. This was different from previous results showing no involvement of *omcZ* in reduction of Fe(III) oxides (Nevin et al., 2009). Although it remains unclear, this apparent discrepancy maybe attributed to the different bacterial strains and experimental conditions used. Compared to that of single-gene-deletion mutants, the abilities to reduce ferrihydrite of the mutants deficient in more than one genes were lower. Deletion of all these genes nearly abolished the bacterial ability to reduce ferrihydrite, which demonstrate the essential roles of these proteins in extracellular reduction of ferrihydrite by *G. sulfurreducens*.

Involvement of *pilA-N* in biofilm formation on nonconductive surface by *G. sulfurreducens* was demonstrated previously (Reguera et al., 2007; Richter et al., 2009, 2012). Over expressions of *pilA-N, omcS, omcT, omcZ* or *omcS* and *omcT* also increased biofilm formation on non-conductive surface (Wang et al., 2023). Our results were consistent with these previous observations. They also showed the involvements of *OmcE* in biofilm formation on non-conductive surface. Thus, *PilA-N* and all tested *c*-Cyts have roles in biofilm formation on nonconductive surface. Notably, the ability to form biofilm on non-conductive surface by Δ*pilA-N* was lower than that of Δ*omcE*, Δ*omcS*, Δ*omcT*, Δ*omcZ* and Δ*omcS*Δ*omcT*, which is consistent with the proposed roles of *PilA-N* in extracellular secretions of *OmcS* and *OmcZ* (Gu et al., 2021).

Previous results showed that deletions of *pilA-N* or *omcZ* of *G. sulfurreducens* severely diminished bacterial ability to produce electricity on anodes, while deletions of *omcS* or *omcE* only slightly decreased bacterial ability to produce electricity (Nevin et al., 2009; Richter et al., 2009). Over expressions of *pilA-N*, *omcS*, *omcT*, *omcZ* or *omcS* and *omcT* also increased electricity production on anodes (Wang et al., 2023). Our results not only were consistent with these observations, but also showed that the output voltage on anodes by Δ*omcS* during first 60-h growth on anodes was trivial, which show the importance of *OmcS* in the early stage of growth on anodes. After that, the output voltage on anodes by Δ*omcS* increased rapidly and then plateaued at 85-h after growth, which suggest that Δ*omcS* may regain its ability to grow on anodes though compensatory roles of other multiheme *c*-Cyts, such as *OmcZ* (Yalcin et al., 2020; Yalcin and Malvankar, 2020). Deletion of *omcT* also severely diminished bacterial ability to produce electricity. Notably, the electricity production by Δ*pilA-N* was similar to that of Δ*omcS*Δ*omcT*Δ*omcZ*, but lower than that of Δ*omcT*, Δ*omcZ* and Δ*omcS*Δ*omcT*, which demonstrate a more dominant role of PilA-N in electricity production than that OmcZ as well as that of OmcS and OmcT combined. The observed dominant roles of PilA-N in electricity production and biofilm formation on nonconductive surface are consistent with its proposed function in extracellular translocation of OmcS and OmcZ across the outer membrane (Gu et al., 2021). Finally, deletion of *pilA-N* and all tested *c*-Cyt genes nearly abolished bacterial ability to produce electricity and to form biofilm on anodes, which also show the essential roles of PilA-N and tested *c*-Cyts in extracellular respiration of anodes.

Previous results showed the involvements of *pilA-N* and *omcS* of *G. sulfurreducens* in the co-culture of *G. metallireducens* and *G. sulfurreducens* and growth improvements of the co-culture by deleting *hybL* gene *G. sulfurreducens* (Summers et al., 2010). Our results are consistent with these results. Additionally, our results also showed that compared to that of WT and Δ*hybL*, ability of the Δ*omcT* and Δ*omcZ* with or without *hybL* to form the co-culture with *G. metallireducens* was impaired, which show for the first time the involvements of OmcT and OmcZ in the DIET from *G. metallireducens* to *G. sulfurreducens*. Although Δ*omcE* exhibited similar ability of co-culture with *G. metallireducens* to that of WT, Δ*omcE*Δ*hybL* showed slightly decreased ability (*p* ≤ 0.001) to co-culture with *G. metallireducens* as compared to that Δ*hybL*, which also demonstrate for the first time the involvement of OmcE in the DIET from *G. metallireducens* to *G. sulfurreducens*.

In the presence of *hybL*, deletions of any single *c*-Cyt gene or both *omcS* and *omcT* did not abolish bacterial ability to co-culture with *G. metallireducens*. Deletion of all *omcS*, *omcT* and *omcZ* nearly abolished the ability to co-culture. However, in the absence of *hybL*, deletion of *pilA-N*, *omcZ* or both *omcS* and *omcT* abolished the ability to co-culture, which clearly demonstrate the essential role of PilA-N, OmcZ and both OmcS and OmcT in the DIET from *G. metallireducens* to *G. sulfurreducens*. Requirement of both OmcS and OmcT in the DIET from *G. metallireducens* to *G. sulfurreducens* suggests an overlapping role of these two multiheme *c*-Cyts in the DIET. Finally, our results also show that H_2_-mediated indirect electron transfer may overshadow the roles of *c*-Cyts in the DIET from *G. metallireducens* to *G. sulfurreducens*.

## Conclusion

Our results show the importance of PilA-N, OmcE, OmcS, OmcT and OmcZ of *G. sulfurreducens* in biofilm formation on nonconductive surface, extracellular reduction of ferrihydrite and anodes as well as the DIET from *G. metallireducens* to *G. sulfurreducens*. However, substantial differences were observed in the contributing roles of these extracellular proteins for *G. sulfurreducens* in these processes. Although it plays significant roles in ferrihydrite reduction and biofilm formation on nonconductive surface, OmcE only plays minor roles in anode reduction and the DIET from *G. metallireducens* to *G. sulfurreducens*. Different from OmcE, OmcS plays a crucial role in ferrihydrite reduction, the early stage of anode reduction, biofilm formation on nonconductive surface and the DIET from *G. metallireducens* to *G. sulfurreducens*. But, its role in the late stage of anode reduction is dispensable. PilA-N, OmcT and OmcZ, however, all play critical roles in reduction of ferrihydrite and anodes, biofilm formation on nonconductive surface and the DIET from *G. metallireducens* to *G. sulfurreducens*. The results from this investigation suggest that possession of PilA-N and multiple c-Cyts of different structures enables *G. sulfurreducens* to mediate EET efficiently with substrates of different properties. One of future researches should focus on whether these tested extracellular proteins form conductive filaments under the conditions investigated in this study.

## Data availability statement

The original contributions presented in the study are included in the article/Supplementary material, further inquiries can be directed to the corresponding author.

## Author contributions

LS designed the experiment and acquired funding. JJ, PH, and YL performed the experiment. ZP and LQ developed experimental method. YJ, YH, XD, YD, and LS analyzed the data and prepared manuscript. All authors contributed to the article and approved the submitted version.
