# Supplementary material for: The varied roles of pilA-N, omcE, omcS, omcT, and omcZ in extracellular electron transfer by Geobacter sulfurreducens
Source: Front Microbiol. 2023 Oct 10;14:1251346. doi: 10.3389/fmicb.2023.1251346 (PMC10597711; doi:10.3389/fmicb.2023.1251346)
Supplement: Supplementary file 1 [file Data_Sheet_1.docx]

## The varied roles of *pilA-N*, *omcE*, *omcS*, *omcT* and *omcZ* in extracellular electron transfer by *Geobacter sulfurreducens*

Jie Jiang^1#^，Pengcheng He^1#^, Ying Luo^1#^，Zhaofeng Peng^1^, Yongguang Jiang^1^, Yidan Hu^1^, Lei Qi^2^, Xiuzhu Dong^2^, Yiran Dong^1,3,4,5^, Liang Shi^1,3,4,5*^

^1^ School of Environmental Studies, China University of Geosciences-Wuhan, Wuhan, China

^2^ State Key Laboratory of Microbial Resources, Institute of Microbiology, Chinese Academy of Sciences, Beijing, China

^3^ State Key Laboratory of Biogeology and Environmental Geology, China University of Geosciences-Wuhan, Wuhan, China

^4^State Environmental Protection Key Laboratory of Source Apportionment and Control of Aquatic Pollution, Ministry of Ecology and Environment, China University of Geosciences-Wuhan, Wuhan, China

^5^Hubei Key Laboratory of Yangtze Catchment Environmental Aquatic Science, China University of Geosciences-Wuhan, Wuhan, China

17 pages

7 tables

5 figures.

**TABLE S1.Bacterial strains used in this study**

| Strains | Relevant characteristics | Source |
| --- | --- | --- |
| *Geobacter metallireducens* GS-15 | | |
| *G. metallireducens* GS-15 | Wild type(WT) | ATCC |
| *Geobacter sulfurreducens* PCA | | |
| *G. sulfurreducens* PCA | Wild type (WT) | ATCC |
| HBC1 | Deletion of *hybL* gene from WT *G. sulfurreducens* genome | This study |
| JIJ1 | Deletion of *omcS* gene from WT *G. sulfurreducens* genome | This study |
| JIJ2 | Deletion of *omcS and hybL* genes from WT *G. sulfurreducens* genome | This study |
| JIJ3 | Deletion of *omcT* gene from WT *G. sulfurreducens* genome | This study |
| JIJ4 | Deletion of *omcT and hybL* genes from WT *G. sulfurreducens* genome | This study |
| JIJ5 | Deletion of *omcZ* gene from WT *G. sulfurreducens* genome | This study |
| JIJ6 | Deletion of *omcZ and hybL* genes from WT *G. sulfurreducens* genome | This study |
| JIJ7 | Deletion of *omcE* gene from WT *G. sulfurreducens* genome | This study |
| JIJ8 | Deletion of *omcE and hybL* genes from WT *G. sulfurreducens* genome | This study |
| JIJ9 | Deletion of *pilA*-N gene from WT *G. sulfurreducens* genome | This study |
| JIJ10 | Deletion of *pilA*-N *and hybL* genes from WT *G. sulfurreducens* genome | This study |
| JIJ11 | Deletion of *omcS and omcT* genes from WT *G. sulfurreducens* genome | This study |
| JIJ12 | Deletion of *omcS, omcT* and *hybL* genes from WT *G. sulfurreducens* genome | This study |
| JIJ13 | Deletion of *omcS, omcT* and *omcZ* genes from WT *G. sulfurreducens* genome | This study |
| JIJ14 | Deletion of *omcS, omcT, omcZ* and *hybL* genes from WT *G. sulfurreducens* genome | This study |
| JIJ15 | Deletion of *omcS, omcT, omcZ* and *omcE* genes from WT *G. sulfurreducens* genome | This study |
| JIJ16 | Deletion of *omcS, omcT, omcZ, omcE* and *hybL* genes from WT *G. sulfurreducens* genome | This study |
| JIJ17 | Deletion of *omcS, omcT, omcZ, omcE* and *pilA-*N genes from WT *G. sulfurreducens* genome | This study |
| JIJ18 | Deletion of *omcS, omcT, omcZ, omcE, pilA*-N and *hybL* genes from WT *G. sulfurreducens* genome | This study |
| JIJ19 | mutant Δ*omcS* carrying complement p*omcS* | This study |
| JIJ20 | mutant Δ*omcT* carrying complement p*omcT* | This study |
| JIJ21 | mutant Δ*omcZ* carrying complement p*omcZ* | This study |
| JIJ22 | mutant Δ*omcE* carrying complement p*omcE* | This study |
| JIJ23 | mutant Δ*pilA*-N carrying complement p*pilA* | This study |
| JIJ24 | mutant WT *G. sulfurreducens* carrying the empty vector pBBR1MCS-5 | This study |
| JIJ25 | mutant Δ*omcS* carrying the empty vector pBBR1MCS-5 | This study |
| JIJ26 | mutant Δ*omcT* carrying the empty vector pBBR1MCS-5 | This study |
| JIJ27 | mutant Δ*omcZ* carrying the empty vector pBBR1MCS-5 | This study |
| JIJ28 | mutant Δ*omcE* carrying the empty vector pBBR1MCS-5 | This study |
| JIJ29 | mutant Δ*pilA*-N carrying the empty vector pBBR1MCS-5 | This study |
| ***Escherichia coli*** | | |
| DH5α | Cloning host | Thermo Fisher Scientific |

**TABLE S2. Plasmids used in this study**

| Plasmids | Relevant characteristics | Source or reference |
| --- | --- | --- |
| pCM158 | Cre recombinase expression vector; *Km^r^* | Marx, C. J., et al. 2002 |
| pHPC1 | Cre recombinase expression vector; *Gm^r^* | This study |
| pBBR1MCS-5 | Empty vector | Kovach et al. 1995 |
| pHPC2 | pUC19 carrying kanamycin resistance cassette flanked by *loxP* sites | This study |
| pHPC3 | pUC19 carrying spectinomycin resistance cassette flanked by *loxP* sites | This study |
| pHPC5 | pUC19 carrying the sequence of G. *metallireducens* 16S rRNA gene | This study |
| pHPC6 | pUC19 carrying the sequence of G. *sulfurreducens* 16S rRNA gene | This study |
| pJIJ1 | The *omcS* gene cloned in pBBR1MCS-5 | This study |
| pJIJ2 | The *omcT* gene cloned in pBBR1MCS-5 | This study |
| pJIJ3 | The *omcE* gene cloned in pBBR1MCS-5 | This study |
| pJIJ4 | The *omcZ* gene cloned in pBBR1MCS-5 | This study |
| pJIJ5 | The *pilA*-N gene cloned in pBBR1MCS-5 | This study |

**TABLE S3. Oligonucleotide primers used in this study**

| Primers | Sequence(5’-3’） | Purpose |
| --- | --- | --- |
| *omcS*-UF | AGCTTTACGTGGGTCAGCTC | Construction of Δ*omcS* |
| *omcS*-UR | GGTGGCAGTTCAGACAGGAC | Construction of Δ*omcS* |
| *omcS*-DF | CGCTTCAACCTGGCATACGA | Construction of Δ*omcS* |
| *omcS*-DR | CGTCCCCTGGAGCAGATAAG | Construction of Δ*omcS* |
| *omcS*-LF | GTCCTGTCTGAACTGCCACCCAATAACGAGGAGGA | Construction of Δ*omcS* |
| *omcS*-LR | TCGTATGCCAGGTTGAAGCGCGCTCCCCGCCATGG | Construction of Δ*omcS* |
| YZ-*omcS*-F | CCAGTGAGGTGATCCGGTAG | Validation of Δ*omcS* |
| YZ-*omcS*-R | TCCGGAAGGGAACTACGTCA | Validation of Δ*omcS* |
| HB-*omcS*-F | GGACTAGTGCGCCGGGCAAGTTCGGAGT | Coding sequence of *omcS* |
| HB-*omcS*-R | CGGAATTCTTAGTCCTTGGCGTGGCACT | Coding sequence of *omcS* |
| *omcT*-UF | CGCTTCAACCTGGCATACGA. | Construction of Δ*omcT* |
| *omcT*-UR | CGTCCCCTGGAGCAGATAAG | Construction of Δ*omcT* |
| *omcT*-DF | GCGTACTATGGAAGACCCCC | Construction of Δ*omcT* |
| *omcT*-DR | GCGGGCATCAGGGAATAGAG | Construction of Δ*omcT* |
| *omcT*-LF | CTTATCTGCTCCAGGGGACGCAATAACGAGGAGGA | Construction of Δ*omcT* |
| *omcT*-LR | GGGGGTCTTCCATAGTACGCCGCTCCCCGCCATGG | Construction of Δ*omcT* |
| YZ-*omcT*-F | GTAGAATCCGGCCCCAAAAC | Validation of Δ*omcT* |
| YZ-*omcT*-R | GATCCCCACGGGAAGTATCG | Validation of Δ*omcT* |
| HB-*omcT*-F | GGACTAGTGAAGTCCGGCGACCTGACCG | Coding sequence of *omcT* |
| HB-*omcT*-R | CGGAATTCTCAGTCTTTGGCATGACACT | Coding sequence of *omcT* |
| *omcZ*-UF | CGGTCAGATAGGTGCTGCTC | Construction of Δ*omcZ* |
| *omcZ*-UR | CATTGCTGCGCCGGTTAAG | Construction of Δ*omcZ* |
| *omcZ*-DF | AGACCCTTACCATTACCGGC | Construction of Δ*omcZ* |
| *omcZ*-DR | CCGTGCCGATAGACCTTCTC | Construction of Δ*omcZ* |
| *omcZ*-LF | CTTAACCGGCGCAGCAATGCAATAACGAGGAGGA | Construction of Δ*omcZ* |
| *omcZ*-LR | GCCGGTAATGGTAAGGGTCTCGCTCCCCGCCATGG | Construction of Δ*omcZ* |
| YZ-*omcZ*-F | CGGCGATAATGTCGCTCAAC | Validation of Δ*omcT* |
| YZ-*omcZ*-R | ATGACGGGCATTGGCATTCT | Validation of Δ*omcT* |
| HB-*omcZ*-F | GGACTAGTCTAGTTATATGCAGTAAAAT | Coding sequence of *omcZ* |
| HB-*omcZ*-R | CGGAATTCTTACCGTTTGACTTTCTTCGG | Coding sequence of *omcZ* |
| *omcE*-UF | GCTGCTTGTGTCGACTTGTGC | Construction of Δ*omcE* |
| *omcE*-UR | GGTCGGTGTTCGTCGCCTTG | Construction of Δ*omcE* |
| *omcE*-DF | CACCCTCTACTCGTCCAGC | Construction of Δ*omcE* |
| *omcE*-DR | TCATGGAACCCACGAATCAC | Construction of Δ*omcE* |
| *omcE*-LF | CAAGGCGACGAACACCGACCCAATAACGAGGAGGA | Construction of Δ*omcE* |
| *omcE*-LR | GCTGGACGAGTAGAGGGTGCGCTCCCCGCCATGG | Construction of Δ*omcE* |
| YZ-*omcE*-F | GACGGGAAGTTTCTCCGCAA | Validation of Δ*omcE* |
| YZ-*omcE*-R | ACGGAGGGGTTGTAGTTGGT | Validation of Δ*omcE* |
| HB-*omcE*-F | GGACTAGTGCTGCTTGTGTCGACTTGTG | Coding sequence of *omcE* |
| HB-*omcE*-R | CGGAATTCCTACTTCTTGTGGCAACCCA | Coding sequence of *omcE* |
| *pilA*-N-UF | GCCGCCTCCAATCGTGATTTG | Construction of Δ*pilA*-N |
| *pilA*-N-UR | TCACTCCTCATCCATGCCAAAT | Construction of Δ*pilA*-N |
| *pilA*-N-DF | GATGATCAAACCTATCCGCCCGA | Construction of Δ*pilA*-N |
| *pilA*-N-DR | ACCAGATGTTGCACCGCGGT | Construction of Δ*pilA*-N |
| *pilA*-N-LF | ATTTGGCATGGATGAGGAGTGACAATAACGAGGAGGA | Construction of Δ*pilA*-N |
| *pilA*-N-LR | TCGGGCGGATAGGTTTGATCATCCGCTCCCCGCCATGG | Construction of Δ*pilA*-N |
| YZ-*pilA*-N-F | TCCTCATGACGGCCTTTTCC | Validation of Δ*pilA*-N |
| YZ-*pilA*-N-R | TGGCCACAATAGCTTGCTGA | Validation of Δ*pilA*-N |
| HB-*pilA*-N-F | GGACTAGTAGTTTTTGGTATTGACAGCA | Coding sequence of Δ*pilA*-N |
| HB-*pilA*-N-R | CCATCGATCTACTGCGACTTCCACTCGG | Coding sequence of Δ*pilA*-N |
| pBBR1MCS-5-F | GTAAAACGACGGCCAGTGAG | Validation of empty vector |
| pBBR1MCS-5-R | AGCTCACTCATTAGGCACCC | Validation of empty vector |
| Gme-F-TA | AAGACCTACGGGGCAAAAGT | Coding sequence of *G. metallireducens* 16S |
| Gme-R-TA | TGGCTGATGCTGGGAATTGA | Coding sequence of *G. metallireducens* 16S |
| Gsu-F-TA | GCAAGCCTCAGCACGAAAC | Coding sequence of *G. sulfurreducens* 16S |
| Gsu-R-TA | CAAGAAGATCAGCACCACCG | Coding sequence of *G. sulfurreducens* 16S |
| Gme-F | ATGGCCCACATCTTCATCTC | *G. metallireducens*quantification； |
| Gme-R | TGCATGTTTTCATCCACGAT | *G. metallireducens*quantification； |
| Gsu-F | CCAGCTACGCCTACTTCTTCTTT | *G. sulfurreducens* quantification |
| Gsu-R | AAGCTGTGGTTCAGGAGGTATTT | *G. sulfurreducens* quantification |

**TABLE S4. Pairwise analyses of Fe(II) formed at 15 days of ferrihydrite reduction by the wild type (WT) and gene-deletion mutants of *Geobacter sulfurreducens*^#^.**

|  | WT | Δ1 | Δ2 | Δ3 | Δ4 | Δ5 | Δ6 | Δ7 | Δ8 | Δ9 |
| --- | --- | --- | --- | --- | --- | --- | --- | --- | --- | --- |
| WT |  |  |  |  |  |  |  |  |  |  |
| Δ1 | *** |  |  |  |  |  |  |  |  |  |
| Δ2 | *** | * |  |  |  |  |  |  |  |  |
| Δ3 | *** | ns | ns |  |  |  |  |  |  |  |
| Δ4 | *** | * | * | * |  |  |  |  |  |  |
| Δ5 | *** | ns | ns | ns | * |  |  |  |  |  |
| Δ6 | *** | *** | *** | *** | ns | *** |  |  |  |  |
| Δ7 | *** | *** | *** | *** | ns | *** | * |  |  |  |
| Δ8 | *** | *** | *** | *** | ns | *** | * | ns |  |  |
| Δ9 | *** | *** | *** | *** | ns | *** | ** | * | ** |  |

**^#^**Δ1, Δ*omcS;* Δ2, Δ*omcT*; Δ3, Δ*omcE*; Δ4, Δ*omcZ*; Δ5, Δ*pilA-N;* Δ6, Δ*omcS*Δ*omcT*; Δ7, Δ*omcS*Δ*omcT*Δ*omcZ;* Δ8, Δ*omcS*Δ*omcT*Δ*omcZ*Δ*omcE*; Δ9, Δ*omcS*Δ*omcT*Δ*omcZ*Δ*omcE*Δ*pilA-N*. ns, P > 0.05; *, P ≤ 0.05; **, P ≤ 0.01; ***, P ≤ 0.001.

**TABLE S5. Pairwise analyses of absorbance of crystal violet (OD_565_) extracted from stained biofilms grown on non-conductive surfaces for 72 hours by the wild type (WT) and gene-deletion mutants of *Geobacter sulfurreducens*^#^.**

|  | WT | Δ1 | Δ2 | Δ3 | Δ4 | Δ5 | Δ6 | Δ7 | Δ8 | Δ9 |
| --- | --- | --- | --- | --- | --- | --- | --- | --- | --- | --- |
| WT |  |  |  |  |  |  |  |  |  |  |
| Δ1 | *** |  |  |  |  |  |  |  |  |  |
| Δ2 | *** | ns |  |  |  |  |  |  |  |  |
| Δ3 | ** | ns | ns |  |  |  |  |  |  |  |
| Δ4 | ** | ns | ns | ns |  |  |  |  |  |  |
| Δ5 | *** | ** | ** | * | * |  |  |  |  |  |
| Δ6 | *** | *** | ** | ns | ns | * |  |  |  |  |
| Δ7 | *** | *** | *** | * | * | ns | ** |  |  |  |
| Δ8 | *** | *** | *** | ** | * | ns | ** | ns |  |  |
| Δ9 | *** | *** | *** | ** | ** | * | ** | * | ns |  |

**^#^**Δ1, Δ*omcS;* Δ2, Δ*omcT*; Δ3, Δ*omcE*; Δ4, Δ*omcZ*; Δ5, Δ*pilA-N;* Δ6, Δ*omcS*Δ*omcT*; Δ7, Δ*omcS*Δ*omcT*Δ*omcZ;* Δ8, Δ*omcS*Δ*omcT*Δ*omcZ*Δ*omcE*; Δ9, Δ*omcS*Δ*omcT*Δ*omcZ*Δ*omcE*Δ*pilA-N*. ns, P > 0.05; *, P ≤ 0.05; **, P ≤ 0.01; ***, P ≤ 0.001.

**Table S6. Pairwise analyses of the maximum output currents measured at 110 hours on anodes grown by the wild type (WT) and gene deletion mutants of *Geobacter sulfurreducens*^#^.**

|  | WT | Δ1 | Δ2 | Δ3 | Δ4 | Δ5 | Δ6 | Δ7 | Δ8 | Δ9 |
| --- | --- | --- | --- | --- | --- | --- | --- | --- | --- | --- |
| WT |  |  |  |  |  |  |  |  |  |  |
| Δ1 | ns |  |  |  |  |  |  |  |  |  |
| Δ2 | ** | * |  |  |  |  |  |  |  |  |
| Δ3 | ns | ns | * |  |  |  |  |  |  |  |
| Δ4 | ** | ns | ns | ** |  |  |  |  |  |  |
| Δ5 | *** | ns | ns | *** | * |  |  |  |  |  |
| Δ6 | ** | ns | ns | ** | ns | ns |  |  |  |  |
| Δ7 | *** | * | ns | *** | ** | ns | ** |  |  |  |
| Δ8 | *** | * | * | *** | * | ns | * | ns |  |  |
| Δ9 | *** | ** | * | *** | *** | ns | *** | *** | ns |  |

**^#^**Δ1, Δ*omcS;* Δ2, Δ*omcT*; Δ3, Δ*omcE*; Δ4, Δ*omcZ*; Δ5, Δ*pilA-N;* Δ6, Δ*omcS*Δ*omcT*; Δ7, Δ*omcS*Δ*omcT*Δ*omcZ;* Δ8, Δ*omcS*Δ*omcT*Δ*omcZ*Δ*omcE*; Δ9, Δ*omcS*Δ*omcT*Δ*omcZ*Δ*omcE*Δ*pilA-N*. ns, P > 0.05; *, P ≤ 0.05; **, P ≤ 0.01; ***, P ≤ 0.001.

**Table S7. Paire-wise analyses of maximum copy numbers of combined 16S rRNA genes detected at the second generation of co-cultures between *G. metallireducens* and the wild type (WT) or gene deletion mutants of *Geobacter sulfurreducens*^#^**

|  | WT | Δ1 | Δ2 | Δ3 | Δ4 | Δ5 | Δ6 | Δ7 | Δ8 | Δ9 |
| --- | --- | --- | --- | --- | --- | --- | --- | --- | --- | --- |
| WT |  |  |  |  |  |  |  |  |  |  |
| Δ1 | *** |  |  |  |  |  |  |  |  |  |
| Δ2 | *** | *** |  |  |  |  |  |  |  |  |
| Δ3 | ns | *** | *** |  |  |  |  |  |  |  |
| Δ4 | *** | ** | *** | *** |  |  |  |  |  |  |
| Δ5 | *** | ns | *** | *** | *** |  |  |  |  |  |
| Δ6 | *** | ** | *** | *** | *** | *** |  |  |  |  |
| Δ7 | *** | *** | *** | *** | *** | *** | *** |  |  |  |
| Δ8 | *** | *** | *** | *** | *** | *** | *** | ** |  |  |
| Δ9 | *** | *** | *** | *** | *** | *** | *** | *** | *** |  |

**^#^**Δ1, Δ*omcS;* Δ2, Δ*omcT*; Δ3, Δ*omcE*; Δ4, Δ*omcZ*; Δ5, Δ*pilA-N;* Δ6, Δ*omcS*Δ*omcT*; Δ7, Δ*omcS*Δ*omcT*Δ*omcZ;* Δ8, Δ*omcS*Δ*omcT*Δ*omcZ*Δ*omcE*; Δ9, Δ*omcS*Δ*omcT*Δ*omcZ*Δ*omcE*Δ*pilA-N*. ns, P > 0.05; **, P ≤ 0.01; ***, P ≤ 0.001.

**Table S8. Paire-wise analyses of maximum copy numbers of combined 16S rRNA genes detected at the second generation of co-cultures between *G. metallireducens* and the Δ*hybL*, Δ*omcS/*Δ*hybL*, Δ*omcT*/Δ*hybL* or Δ*omcE*/Δ*hybL* mutants of *Geobacter sulfurreducens*^#^**

|  | Δ*hybL* | Δ1/Δ*hybL* | Δ2/Δ*hybL* | Δ3Δ*hybL* |
| --- | --- | --- | --- | --- |
| Δ*hybL* |  |  |  |  |
| Δ1/Δ*hybL* | *** |  |  |  |
| Δ2/Δ*hybL* | *** | * |  |  |
| Δ3/Δ*hybL* | *** | ** | * |  |

**^#^**Δ1, Δ*omcS*/Δ*hybL;* Δ2/Δ*hybL*, Δ*omcT*/Δ*hybL*; Δ3, Δ*omcE*/Δ*hybL.*


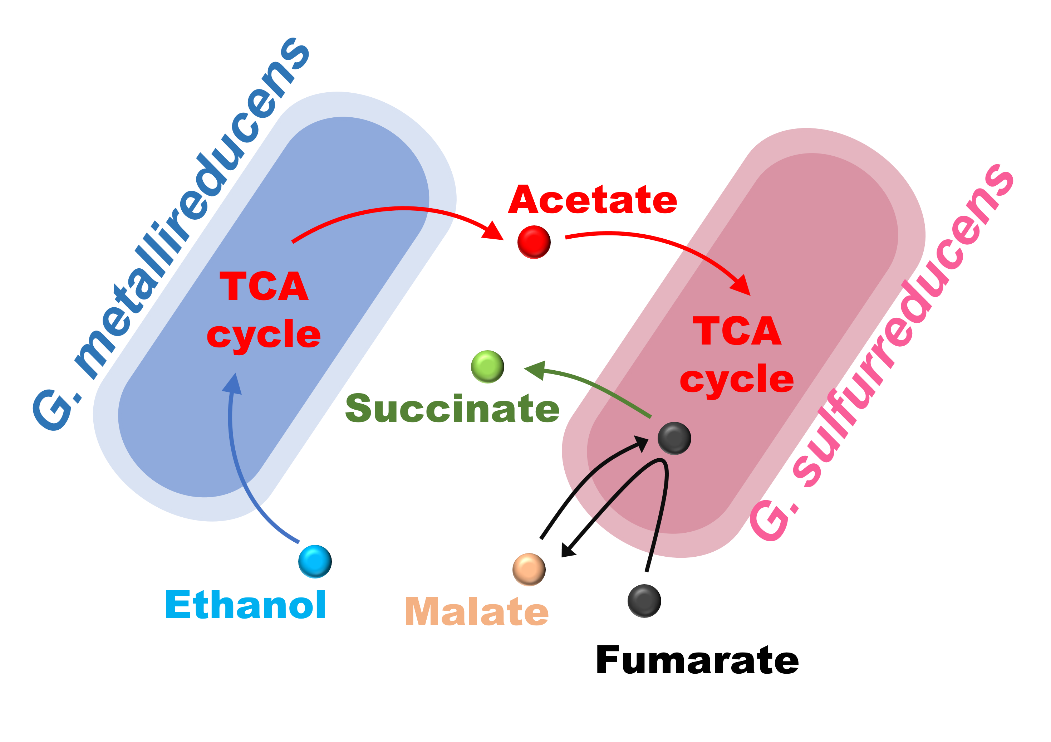


Figure S1. The metabolisms of ethanol, fumarate, malate and succiniate during co-culture of *Geobacter metallireducens* and *Geobacter sulfurreducens*.

**
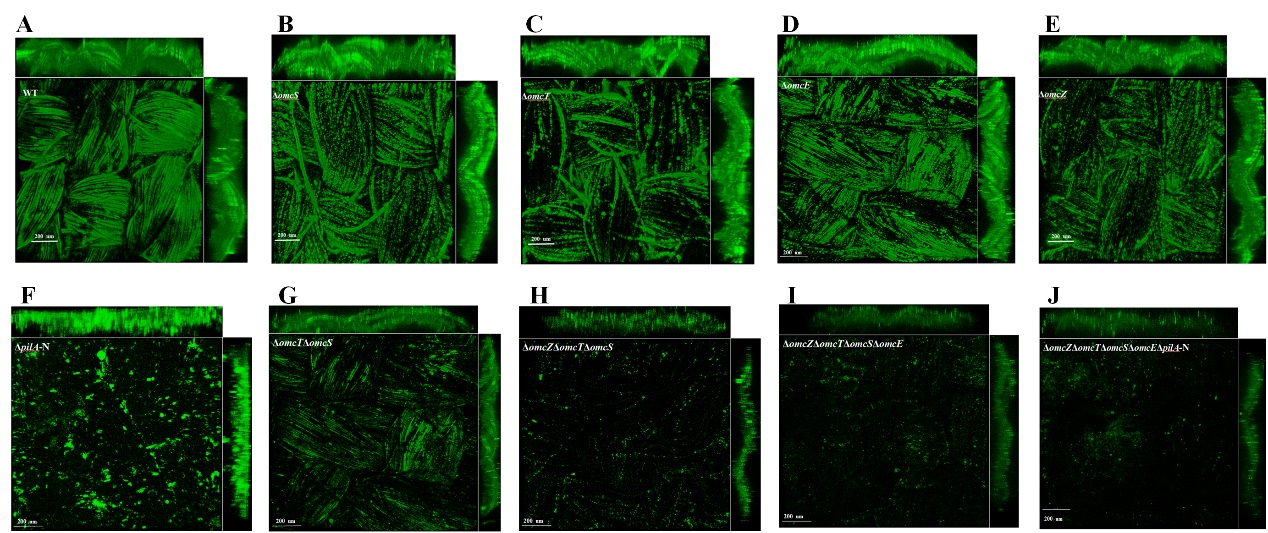
**

Figure S2. Confocal microscopic images of the biofilms of *Geobacter sulfurreducens* strains formed on the anodes at 110 hours after incubation. A. Wild-type. B. Δ*omcS*. C. Δ*omcT*. D. Δ*omcE*. E. Δ*omcZ*. F. Δ*pilA-N*. G. Δ*omcS*Δ*omcT*. H. Δ*omcS*Δ*omcT*Δ*omcZ*. I. Δ*omcS*Δ*omcT*Δ*omcZ*Δ*omcE*. J. Δ*omcS*Δ*omcT*Δ*omcZ*Δ*omcE*Δ*pilA-N*.


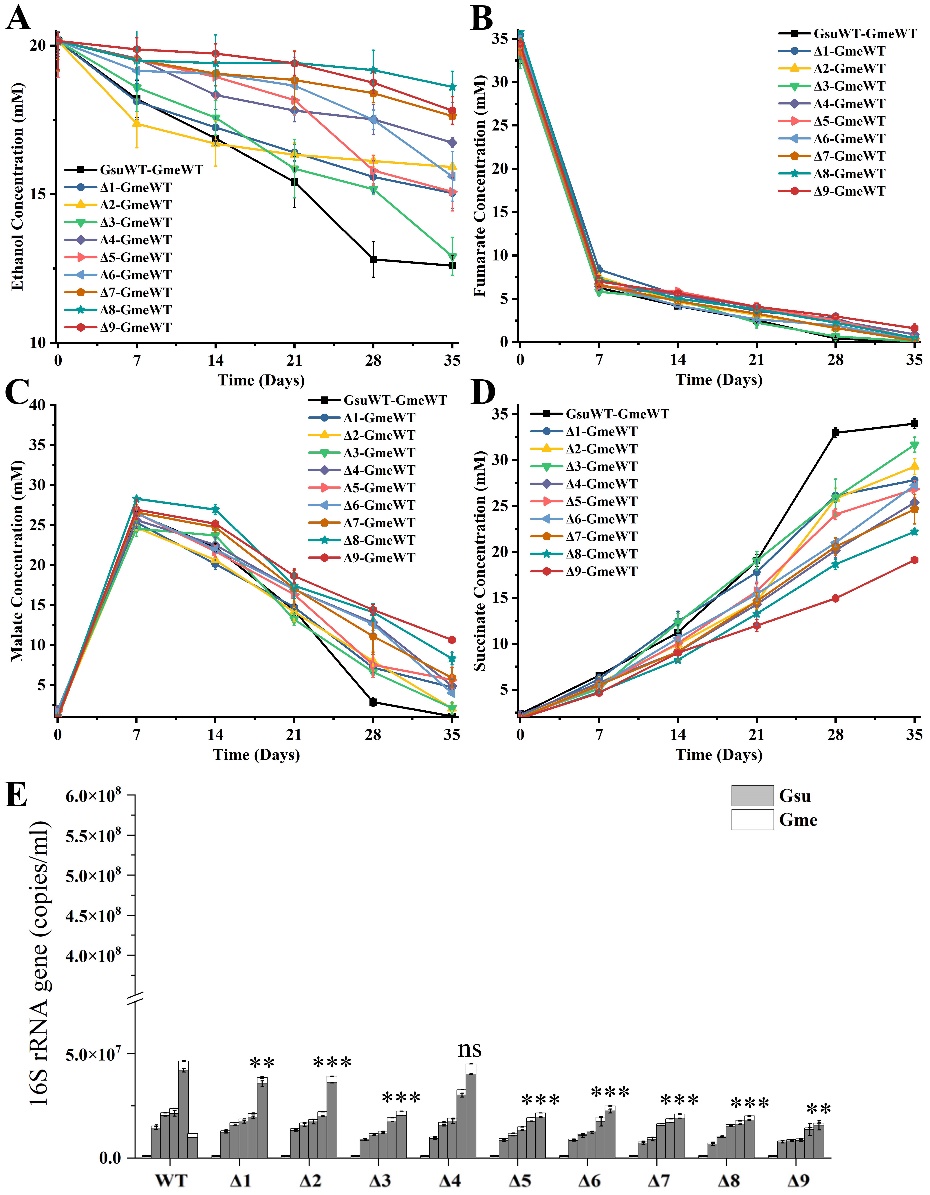


Figure S3. The first generation of co-cultures in the presence of *hybL* gene of *Geobacter sulfurreducens* over 35 days. The values plotted at each time point are the average ethanol (A), fumarate (B), malate (C) and succinate (D) measured for each strain (n = 3), respectively, and error bars are standard deviations. For points with no error bar, the error was smaller than the size of the symbol. E. The copies of combined 16S rRNA genes of *Geobacter metallireducens* (Gme) and *G. sulfurreducens* (Gsu) (n = 3). The predetermined time points for sample collections in E are the same to those in A-D. WT, wild-type of *G. sulfurreducens* or *G. metallireducens*; Δ1, Δ*omcS* of *G. sulfurreducens*; Δ2, Δ*omcT* of *G. sulfurreducens*; Δ3, Δ*omcE* of *G. sulfurreducens*; Δ4, Δ*omcZ* of *G. sulfurreducens*; Δ5, Δ*pilA-N* of *G. sulfurreducens*; Δ6, Δ*omcS*Δ*omcT* of *G. sulfurreducens*; Δ7, Δ*omcS*Δ*omcT*Δ*omcZ* of *G. sulfurreducens*; Δ8, Δ*omcS*Δ*omcT*Δ*omcZ*Δ*omcE* of *G. sulfurreducens*; Δ9, Δ*omcS*Δ*omcT*Δ*omcZ*Δ*omcE*Δ*pilA-N* of *G. sulfurreducens*. In E, Student’s *t* test was used for comparing the maximum copies of combined 16S rRNA genes of Gme and Gus from WT and that of the mutants. ns, P > 0.05; **, P ≤ 0.01; ***, P ≤ 0.001.


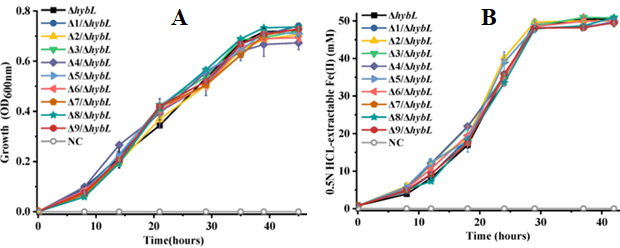
Figure S4. Characterizations of the constructed nanowire mutants of *Geobacter sulfurreducens* without *hybL*. A Growth with fumarate as the sole terminal electron acceptor. B. Fe(III)-citrate reduction. Δ*hybL*, Δ*hybL* of *G. sulfurreducens*; Δ1/Δ*hybL*, Δ*omcS* of Δ*hybL*; Δ2/Δ*hybL*, Δ*omcT* of Δ*hybL*; Δ3/Δ*hybL*, Δ*omcE* of Δ*hybL*; Δ4/Δ*hybL*, Δ*omcZ* of Δ*hybL*; Δ5/Δ*hybL*, Δ*pilA-N* of Δ*hybL*; Δ6/Δ*hybL*, Δ*omcS*Δ*omcT* of Δ*hybL*; Δ7/Δ*hybL*, Δ*omcS*Δ*omcT*Δ*omcZ* of Δ*hybL*; Δ8/Δ*hybL*, Δ*omcS*Δ*omcT*Δ*omcZ*Δ*omcE* of Δ*hybL*; Δ9/Δ*hybL*, Δ*omcS*Δ*omcT*Δ*omcZ*Δ*omcE*Δ*pilA-N* of Δ*hybL*; NC, no cell control. The values plotted at each time point are the average OD_600_ (A) and 0.5 N HCl extractable Fe(II) (B) measured for each strain (n = 3), respectively, and error bars are standard deviations. For points with no error bar, the error was smaller than the size of the symbol.


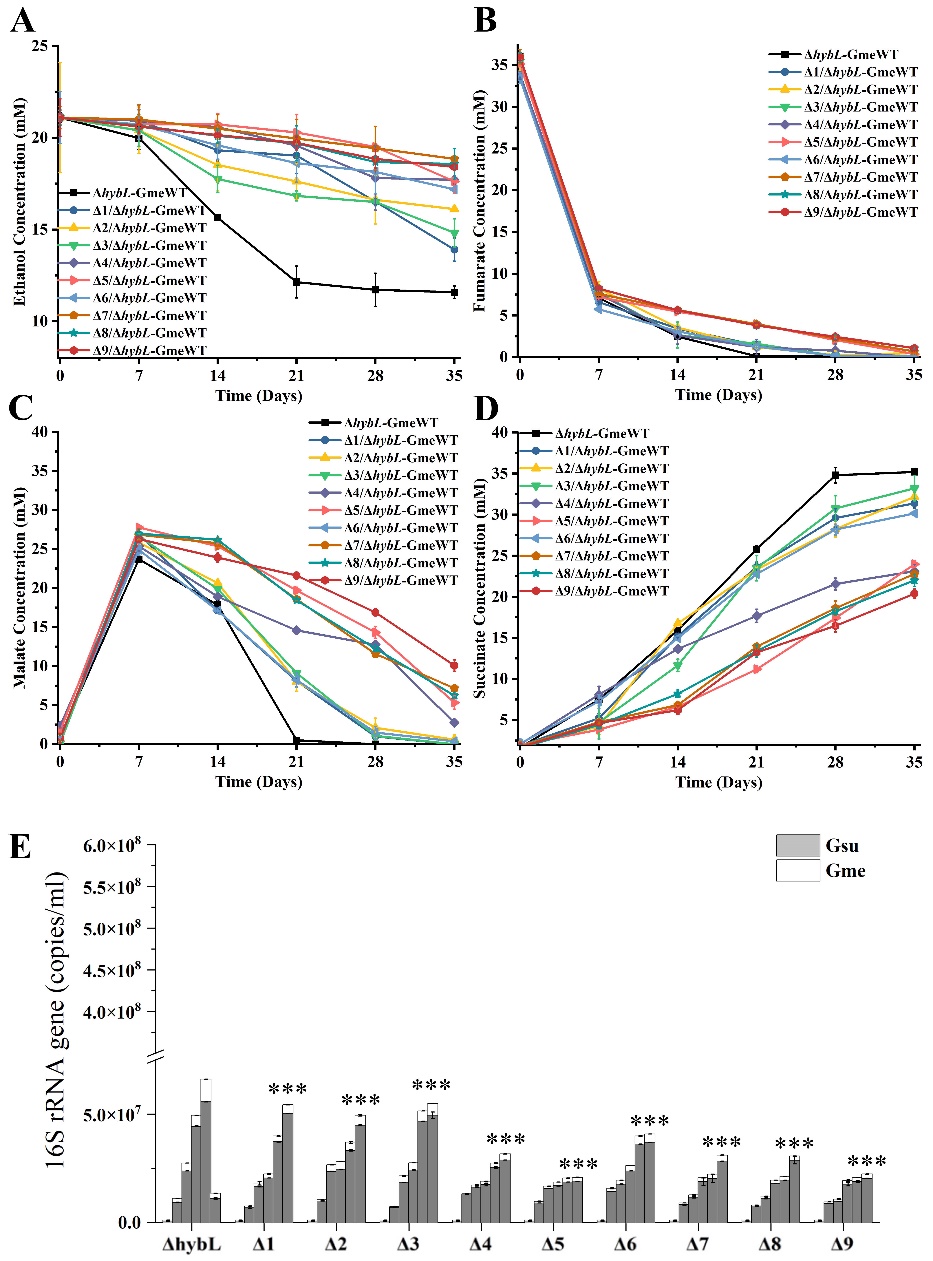


Figure S5. The first generation of co-cultures in the absence of *hybL* gene of *Geobacter sulfurreducens* over 35 days. The values plotted at each time point are the average ethanol (A), fumarate (B), malate (C) and succinate (D) measured for each strain (n = 3), respectively, and error bars are standard deviations. For points with no error bar, the error was smaller than the size of the symbol. E. The copies of combined 16S rRNA genes of *Geobacter metallireducens* (Gme) and *G. sulfurreducens* (Gsu) (n = 3). The predetermined time points for sample collections in E are the same to those in A-D. WT, wild-type of *G. metallireducens*; Δ*hybL*, Δ*hybL* of *G. sulfurreducens*; Δ1/Δ*hybL*, Δ*omcS* of Δ*hybL*; Δ2/Δ*hybL*, Δ*omcT* of Δ*hybL*; Δ3/Δ*hybL*, Δ*omcE* of Δ*hybL*; Δ4/Δ*hybL*, Δ*omcZ* of Δ*hybL*; Δ5/Δ*hybL*, Δ*pilA-N* of Δ*hybL*; Δ6/Δ*hybL*, Δ*omcS*Δ*omcT* of Δ*hybL*; Δ7/Δ*hybL*, Δ*omcS*Δ*omcT*Δ*omcZ* of Δ*hybL*; Δ8/Δ*hybL*, Δ*omcS*Δ*omcT*Δ*omcZ*Δ*omcE* of Δ*hybL*; Δ9/Δ*hybL*, Δ*omcS*Δ*omcT*Δ*omcZ*Δ*omcE*Δ*pilA-N* of Δ*hybL*. In E, Student’s *t* test was used for comparing the maximum copies of combined 16S rRNA genes of Gme and Gus from Δ*hybL* and that of other mutants without *hybL*. ns, P > 0.05; *, P ≤ 0.05; **, P ≤ 0.01; ***, P ≤ 0.001.
